# Is Photoluminescence Spectroscopy a Suitable Probe of Halide Segregation?

**DOI:** 10.1021/acsenergylett.6c00432

**Published:** 2026-04-21

**Authors:** Joshua R. S. Lilly, Vincent J.-Y. Lim, Jay B. Patel, Jae Eun Lee, Siyu Yan, Michael B. Johnston, Laura M. Herz

**Affiliations:** † Clarendon Laboratory, Department of Physics, 6396 University of Oxford, Parks Road, Oxford OX1 3PU, United Kingdom; ‡ Department of Physics, King’s College London, Strand, London WC2R 2LS, United Kingdom

## Abstract

Mixed-halide perovskites exhibit ideal band gaps for use in perovskite-based multijunction photovoltaics, but stable performance is compromised by light-induced halide segregation. Photoluminescence (PL) tracking is universally used to monitor such photoinstability; however, here we reveal that such data do not accurately quantify halide segregation. We utilize a combination of simultaneously recorded PL and X-ray diffraction (XRD) measurements to explore CH_3_NH_3_Pb­(I_1–*x*_Br_*x*_)_3_ films across 18 different halide ratios. While PL data suggests that segregation rates increase exponentially with bromide fraction *x*, XRD patterns reveal that they are actually unchanged. We demonstrate that PL cannot accurately reflect the rate and extent of halide segregation because it is governed by charge funneling to iodide-rich minority domains, which is strongly influenced by additional factors, including luminescence efficiency, band energetics, and charge extraction. To assess the efficacy of treatments to suppress such photoinstabilities, it is therefore essential to probe changes across the full material volume, e.g. by monitoring XRD or absorption spectra.

## Graphical abstract

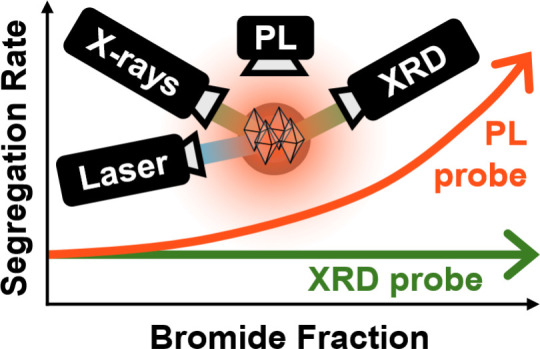

Metal-halide perovskites (MHPs) have generated great excitement in the field of photovoltaics because of their favorable optoelectronic properties.^1–7^ Most significantly, the band gap of MHPs can be conveniently manipulated by alteration of their chemical composition (ABX_3_, where A is a monovalent cation, B is a divalent metal cation, and X is a monovalent halide anion).^8–10^ Such tunability is particularly advantageous to the development of high-efficiency solar cell architectures such as silicon-perovskite and all-perovskite multi junctions, as well as spectrally diverse light-emitting diodes.^11–17^ Multijunction solar cell architectures require a range of absorber materials with carefully chosen band gaps, which can be achieved by alloying iodide and bromide on the halide X-site of MHPs.^11,15^ Unfortunately, under light or electronic bias, iodide- and bromide-enriched regions have been shown to develop in mixed-halide perovskites, causing local variation in band gap across the material.^10,18–22^ Termed ‘halide segregation’, this reversible phenomenon leads to increased radiative losses, reduced charge-carrier diffusion lengths, and hence, diminished photovoltaic performance.^23–25^

As halide segregation forms a pre-eminent barrier to the development of commercially viable perovskite-based solar cells, many investigations have attempted to uncover the underlying physics and develop methodologies to suppress such undesired photoinstability.^13,20,21,26–52^ In such studies, photoluminescence spectroscopy has been the most commonly used technique by far to investigate halide segregation.^25,30–34,37,44,46,47,50–59^ The reason for this choice appears to be its simplicity and its ability to amplify even minor segregation effects through charge funneling. As the material segregates, regions of iodide-rich perovskite are generated whose electronic band gap is narrower than that of the surrounding material. Charge carriers diffusing through the material funnel into such lower-energy domains, leading to a red-shift in photoluminescence (PL) spectra that is easily detected.^21,53,60^ The extent of this red-shift has widely been used as a proxy for the halide segregation dynamics, and in turn to assess whether relative stability can be influenced by changes in composition, processing or treatment of mixed-halide perovskite films.^9,42,44,46,47,51–54^ However, such changes in PL spectra arise from the capture, retention and radiative recombination of charge carriers in iodide-rich domains, which only comprise a small fraction of the total film volume.^19,61^ Given that PL spectroscopy therefore does not reflect changes occurring across the full volume of the material, it is pertinent to ask whether it is actually a valid technique to probe halide segregation. This question is highly critical to address, given that the efficacy of treatments to improve the photostability of candidate materials is currently frequently assessed via photoluminescence measurements.^9,42,44,46,47,51,52^ In comparison, there has been much more limited adoption of techniques that are suitably sensitive to average bulk changes in structure or composition, such as X-ray diffractometry (XRD) and optical absorption spectroscopy.^36,62–64^

In this study, we combine in situ, simultaneously recorded XRD and PL measurements to demonstrate that photoluminescence spectroscopy is not an effective tool for probing halide segregation in mixed-halide MHP thin-films. By investigating mixed lead iodide-bromide perovskite films across the full compositional space, we show that monitoring of PL spectral red-shifts yields very different apparent rates of segregation compared with those observed from structural XRD measurements. We unravel the causes for these discrepancies, and show that they arise from a plethora of other factors affecting the recombination of charge carriers as monitored in PL measurements. We show that PL red-shifts following halide segregation are influenced in a nonlinear way by charge diffusion and capture into iodide-rich domains. In addition, thermal reactivation of charge carriers from the iodide-rich phase back into the mixed phase occurs effectively for compositions with less than 20% bromide fraction, making these erroneously seem photostable when monitored through PL spectra. Moreover, nonradiative and radiative recombination pathways are altered both with halide content and by the nature of extraction pathways in a device structure. As a result, the commonly monitored red-shifts occurring in PL spectra during segregation do not reliably reflect the average material response across the full volume, making them an ineffective probe of halide segregation.

We begin our study by contrasting the observed evolution of halide segregation under illumination as captured simultaneously by in situ photoluminescence spectroscopy and X-ray diffraction (experimental details provided in Section 1 of the Supporting Information). We investigated thin films of MAPb­(I_1–*x*_Br_*x*_)_3_ across 18 bromide fractions (*x*), ranging from *x* = 0 to 1 (MA = CH_3_NH_3_; see Section 2 of the Supporting Information for full details of sample preparation). Films were encapsulated with a layer of poly­(methyl methacrylate) (PMMA) to exclude atmospheric effects, and to prevent permanent material degradation, a low incident X-ray flux and low-intensity laser excitation of 0.91 mW cm^–2^ at a wavelength of 470 nm were used.^32,65,66^

Figure 1 reveals the starkly differing measures of halide segregation produced by either PL or XRD measurements for two example compositions, MAPb­(I_0.575_Br_0.425_)_3_ and MAPb­(I_0.4_Br_0.6_)_3_. Figure 1a and b shows how the normalized photoluminescence spectra for the two films change over time under illumination. The initial PL peak red-shifts, consistent with the formation of iodide-enriched regions of narrower band gap – a hallmark of halide segregation. To allow for a simple comparison of segregation rates deduced from such data, we indicate in Figures 1a and 1b with a dashed white line a “switch-over” time (*t*_PL_), defined as the time taken for the emission intensity from iodide-rich regions to become equal in intensity to that emitted from the original mixed phase. Such a PL “switch-over” time has previously been utilized as a metric for the speed of halide segregation.^53^ Interestingly, comparison between Figures 1a and 1b shows that, according to this PL-based metric, halide segregation rates for MAPb­(I_0.575_Br_0.425_)_3_ appear to be an order of magnitude lower than those observed for MAPb­(I_0.4_Br_0.6_)_3_ under identical illumination conditions, suggesting a strong dependence on bromide content.

**Figure 1:**
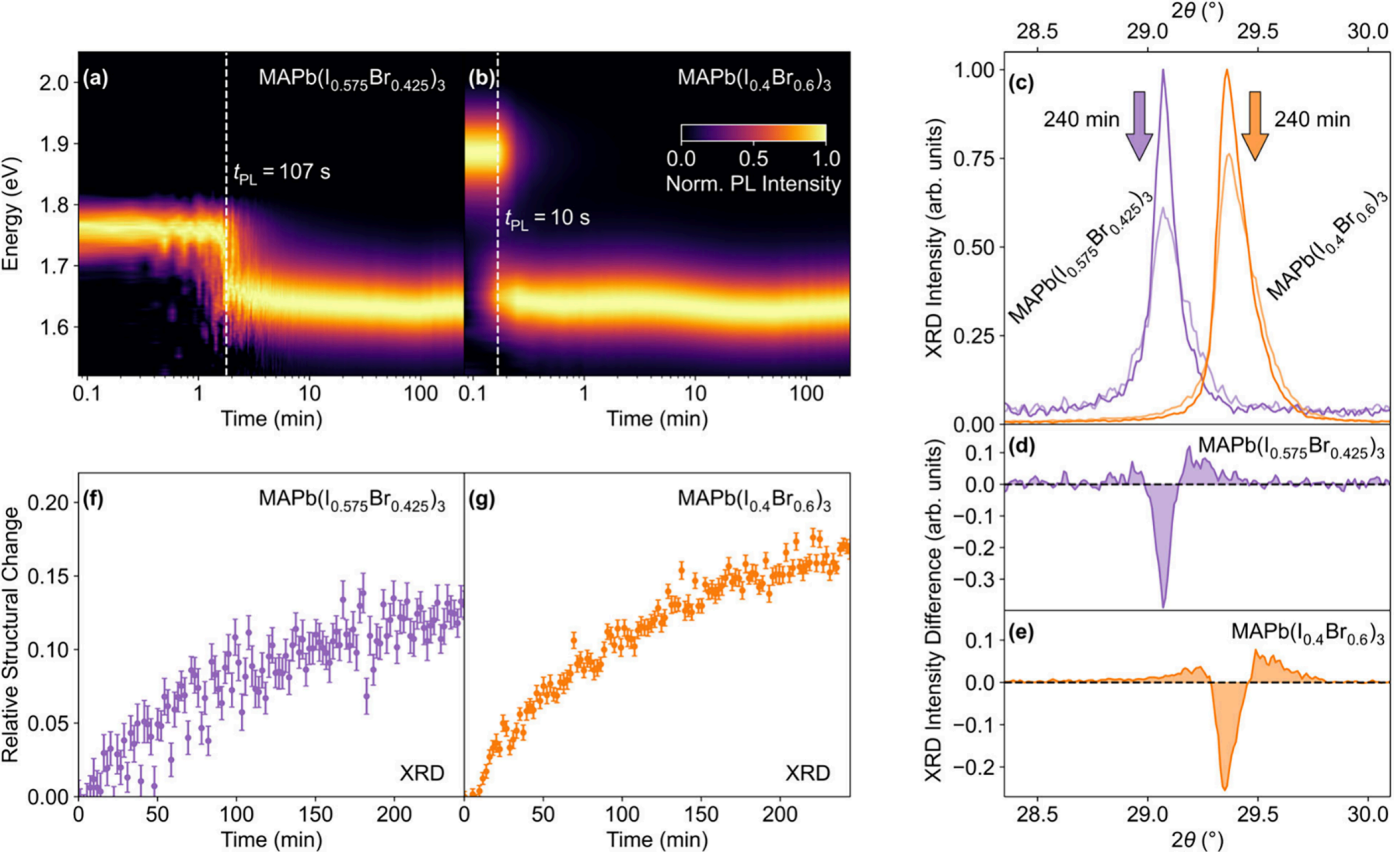
In situ PL and XRD measurements recorded on representative MAPb­(I_0.575_Br_0.425_)_3_ and MAPb­(I_0.4_Br_0.6_)_3_ films encapsulated in PMMA, under illumination with a laser excitation of 0.91 mW cm^–2^ at a wavelength of 470 nm. Diffraction patterns were recorded using monochromatic Cu–Kα_1_ radiation. (a,b) Normalized PL spectra for MAPb­(I_0.575_Br_0.425_)_3_ and MAPb­(I_0.4_Br_0.6_)_3_ films recorded over 4 h of continuous illumination. A moving average window was applied along the wavelength axis to reduce the visual noise level. The “switch-over” time^53^ is indicated with a vertical dashed line. (c) (200) diffraction peak before and after 4 h of continuous illumination for the MAPb­(I_0.575_Br_0.425_)_3_ (purple) and MAPb­(I_0.4_Br_0.6_)_3_ (orange) representative films. (d,e) Intensity difference between the initial (200) diffraction peak and that recorded after 4 h of illumination for the MAPb­(I_0.575_Br_0.425_)_3_ and MAPb­(I_0.4_Br_0.6_)_3_ films. (f,g) Relative structural change occurring in the MAPb­(I_0.575_Br_0.425_)_3_ and MAPb­(I_0.4_Br_0.6_)_3_ films, calculated from the integral over the absolute value of the differential intensity^20^ (see Section 3 of the Supporting Information for further information), e.g., as indicated by the shaded areas in panels (d) and (e).

In contrast, we find that changes in XRD patterns, recorded in situ and concurrently with the PL data discussed above, reveal an entirely different picture. We stress that such XRD measurements, unlike PL data, reflect the structural changes occurring across the whole volume of the material illuminated.^61^ Figure 1c illustrates, again for the example compositions MAPb­(I_0.575_Br_0.425_)_3_ and MAPb­(I_0.4_Br_0.6_)_3_, how the main second-order (200) perovskite diffraction peak changes following 240 min of continuous illumination. Here, the central peak intensity is reduced, reflecting a decrease in the original mixed-halide phase, while higher- and lower-angle wings form indicating that bromide-rich and iodide-rich regions have developed (further analysis is provided in Section 4 of the Supporting Information), as previously observed.^20,61,62^ The total integral across the peak is conserved for both films (Figure S1) reflecting insignificant loss of the overall perovskite material. We derive a metric for the extent of the structural change (see Section 3 of the Supporting Information and ref 20) from the differential intensity plots shown in Figure 1d&e, calculated by subtracting the original diffraction peak from the value at any given time after illumination. By integrating over the absolute value of such differential intensity plots, we can assign a single value to track the structural change induced by halide segregation over time. Intriguingly, as shown in Figure 1f&g, the time evolution of the relative structural change is remarkably similar for MAPb­(I_0.575_Br_0.425_)_3_ and MAPb­(I_0.4_Br_0.6_)_3_ consistent with our previous study.^20^ This finding appears to be in striking disagreement with those derived from red-shifts in the PL data (Figure 1a&b) discussed above, which suggested an order-of-magnitude difference in halide segregation dynamics between MAPb­(I_0.575_Br_0.425_)_3_ and MAPb­(I_0.4_Br_0.6_)_3_. In addition, the photoluminescence dynamics (Figure 1a&b) mostly occur on different time scales (tens of seconds) to those of the structural dynamics (tens of minutes) measured by XRD. This is also evident in the longer-term trends: while the red-shift in PL spectra reaches a steady state within 10 min, the relative structural change in XRD patterns continues to increase beyond the end of the experiment (approximately 4 h) for both compositions.

To further elucidate the origin of such drastic differences in apparent halide segregation dynamics between these types of measurement, we performed in situ concurrent XRD and PL spectroscopy on MAPb­(I_1–*x*_Br_*x*_)_3_ films with 18 different bromide fractions (*x*), illuminating each film for approximately 16 h. Our recent study reporting solely on changes in XRD patterns across this series showed that the volume extent across which halide segregation occurs is symmetric in compositional space, maximizing at approximately *x* = 0.5.^20^ However, the rate of halide segregation is largely invariant across the halide series, consistent with the observation reported in Figure 1f&g above, and likely limited by ionic mobility.^20^ We proceed by correlating such findings with the corresponding PL evolutions taken concurrently and in situ, in order to explain why such spectroscopy alone may not be an appropriate tool to track halide segregation. We quantitatively assess the evolution of the photoluminescence spectra (shown in Figure S2 for each composition) by extracting from each spectrum at a given illumination time an average photon emission energy via a weighted integral.^53^ Figures 2a-c show the resulting plots of average photon energy versus illumination time for a subset of three compositions (see Figure S3 for all other compositions). Consistent with previous reports,^19^ the average photon energy for all compositions (*x* > 0.2) converges to a singular value, approximately 1.65 eV, which reflects emission from a phase with approximately *x* = 0.2 bromide content (Figure S4). However, the rate at which the average photon energy converges on its terminal value varies considerably between compositions, taking approximately 1 h for bromide content *x* = 0.3 (Figure 2a), but only on the order of seconds for *x* = 0.7 (Figure 2c).

**Figure 2:**
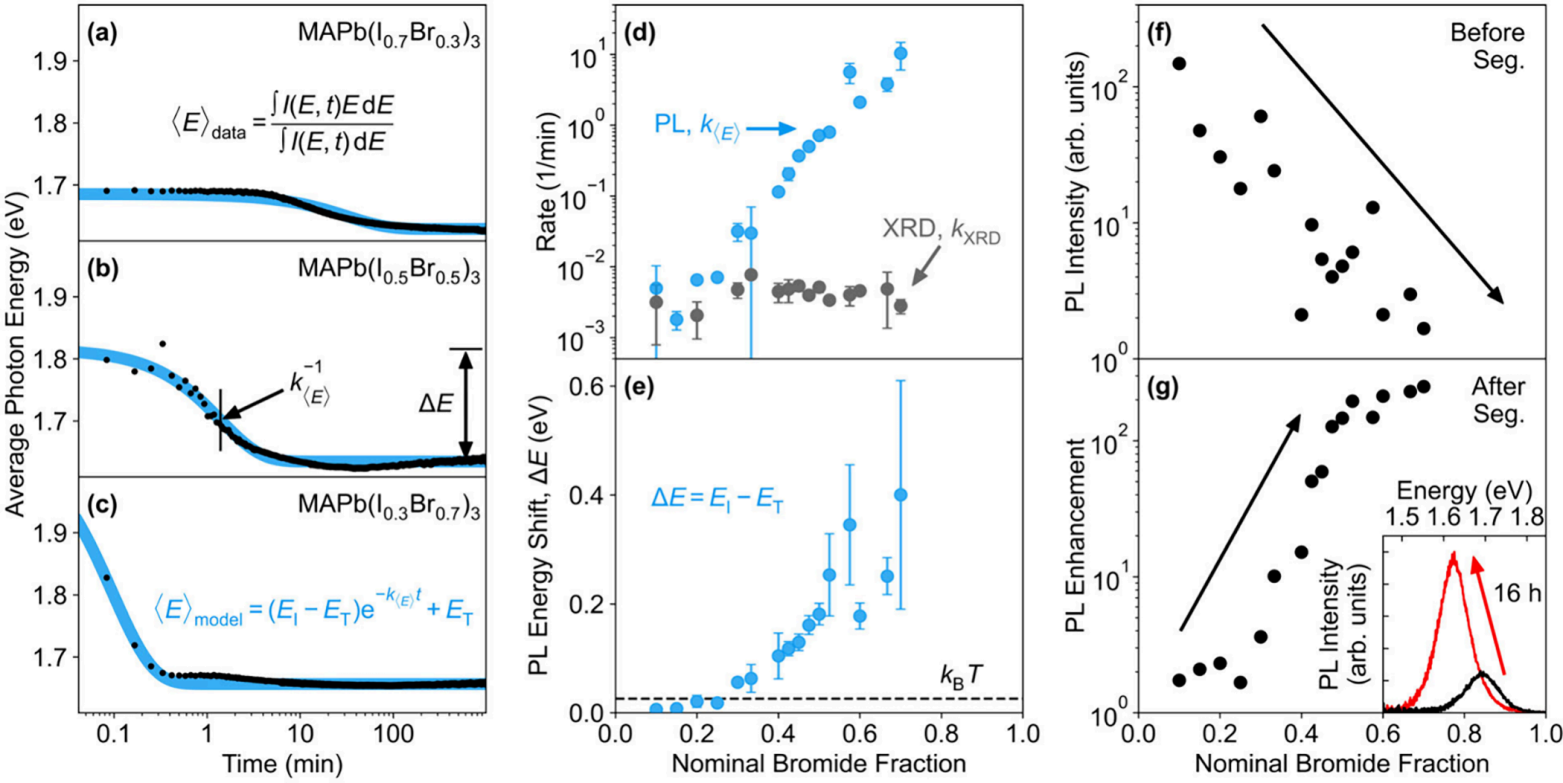
Impact of bromide fraction *x* on halide segregation in films of MAPb­(I_1–*x*_Br_*x*_)_3_ as probed via photoluminescence tracking. Films were encapsulated in PMMA and illuminated for approximately 16 h with a constant laser intensity of 0.91 mW cm^–2^ at a wavelength of 470 nm. (a–c) Evolution of the average photon energy with respect to time for three example compositions. The blue line is a fit to the data based on the equation shown in panel (c). (d) PL-extracted rates in average photon energy shifts, *k*_⟨*E*⟩_, obtained from fits, shown together with XRD-determined rates in structural change, *k*_XRD_, reproduced from ref 20 based on data taken concurrently and in situ. (e) PL energy shifts, Δ*E*, extracted from fits to evolutions of the average photon energy. The dashed black line, marked as *k*_B_T, highlights the energy corresponding to ambient temperature (26 meV). (f) Spectrally integrated intensity of the initial mixed-phase photoluminescence peak (i.e., before segregation). The black arrow is a guide to the eye. >95% of light entering each film is calculated to be absorbed. (g) The maximum increase in the relative spectrally integrated PL intensity, with a black arrow as a guide to the eye. The inset shows an example of such PL enhancement exhibited by a MAPb­(I_0.7_Br_0.3_)_3_ film over 16 h of continuous illumination.

We parametrize such shifts in average photon energy with a simple exponential model function, stated in Figure 2c, which yields both a parameter for the rate of change, *k*_⟨*E*⟩_, and for the total energy change Δ*E* occurring between the initial (*E*_I_) and terminal (*E*_T_) value of the average photon energy. Our model (blue trace, Figure 2a-c, and Figure S3) accurately reproduces the observed dynamics, with the extracted values for *k*_⟨*E*⟩_ and Δ*E* presented in Figures 2d and e. Strikingly, Figure 2d reveals that the PL-derived value for the halide segregation rate (*k*_⟨*E*⟩_) increases exponentially by four orders of magnitude across bromide content *x*, even though the corresponding XRD-derived values (*k*_XRD_)^20^ remain essentially constant. We argue below that while XRD patterns are a sound reflection of the structural changes occurring during halide segregation, red shifts in PL spectra depend on a myriad of additional factors that are only indirectly related to segregation, which may cause erroneous assessments. We proceed by discussing four major factors below, with Figure 3a−d providing schematics to illustrate these effects.

**Figure 3:**
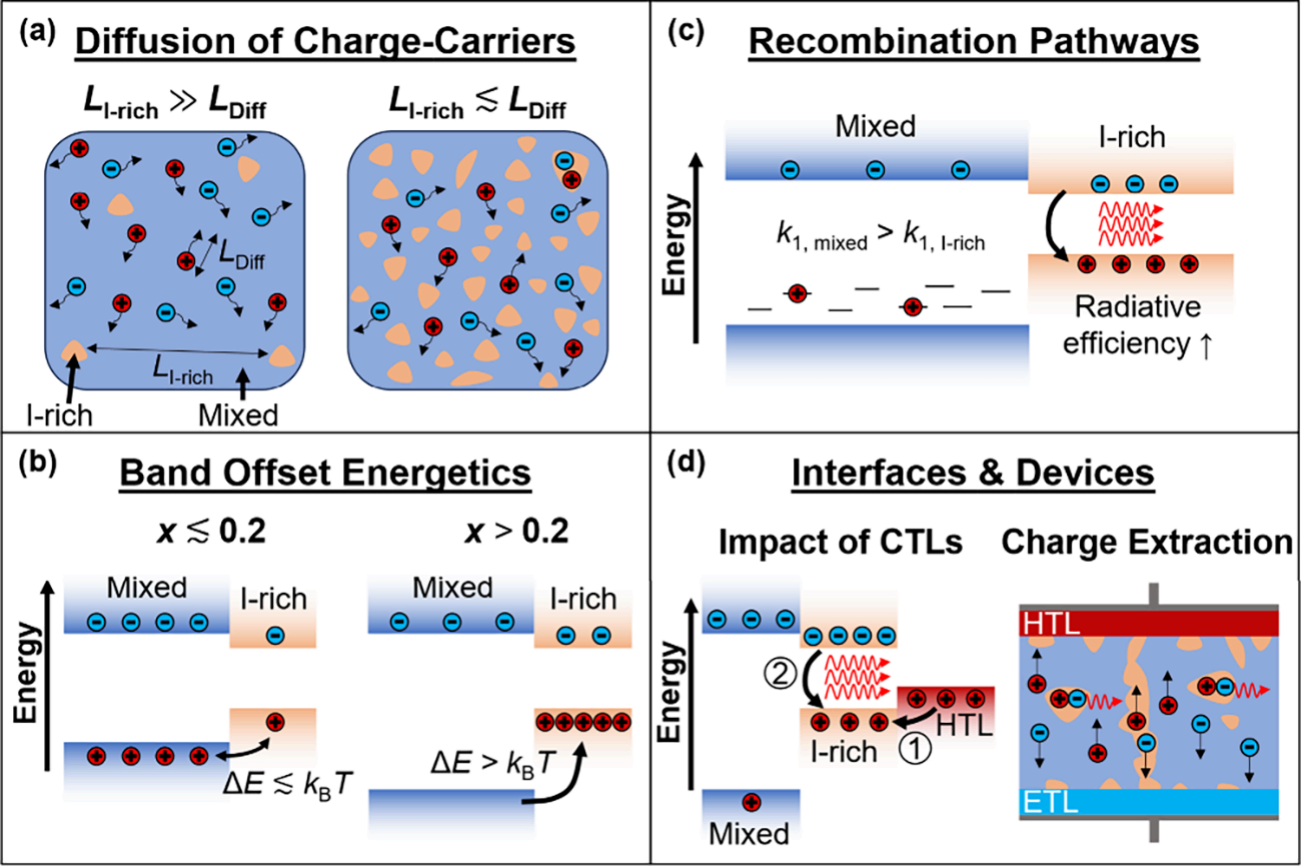
Schematic diagrams describing processes impacting dynamic changes in the PL spectrum of a mixed-halide perovskite undergoing segregation under illumination. Note that bromide-rich regions have been omitted in the schematic for simplicity as they possess a wider band gap and therefore do not meaningfully influence the PL spectrum. (a) When the characteristic length scale separating iodide-rich regions (*L*_I‑rich_) becomes comparable to the charge-carrier diffusion length (*L*_Diff_), charge carriers excited in mixed-phase regions will rapidly diffuse to and recombine in iodide-rich regions. (b) When the energy difference (Δ*E*) between the mixed-phase and iodide-rich phase exceeds the ambient thermal energy (for *x* ≳ 0.2), charge carriers are more likely to become fully localized in iodide-rich regions. (c) The trap-mediated recombination rate (*k*_1_) is lower in the iodide-rich-phase than the mixed phase, and second-order radiative electron–hole recombination is enhanced as a result of charge funneling. (d) Left: in the presence of a charge-transport layer (CTL), e.g., for holes (HTL), halide segregation predominantly occurs at the interface, and hole back-transfer energetically favors iodide-rich domains, leading to enhanced red-shifted PL.^69^ Right: as the charge-extraction efficiency from mixed-phase regions is superior to that from iodide-rich regions in a device (limited percolation pathways), an artificial acceleration in the PL-derived segregation rate can be observed under operational conditions.^33^

First, we argue that the observed red-shifting of PL spectra does not quantify halide segregation accurately because it is strongly influenced by the speed and extent of charge funneling to iodide-rich domains. Charge carriers are initially photoexcited across the full volume of the film and subsequently diffuse through the material. As depicted in Figure 3a, whether such charge carriers are captured into iodide-rich minority domains formed under illumination depends on whether their relative distance falls within the charge diffusion length. Experimental evidence has suggested that this criterion can be reached even if only a relatively small volume fraction of the material has converted to iodide-rich domains. For example, using cathodoluminescence imaging, Bischak et al. showed that iodide-rich domains are separated by around 100 nm, a comparable length scale to that of charge diffusion lengths in prototypical metal halide perovskites (as discussed in Section 5 of the Supporting Information, observations from such spatially resolved measurements are consistent with the conclusions drawn from Figure 2).^5,7,21,67,68^ Similarly, transient optical-pump THz-probe spectroscopy has shown such charge funneling to occur on a time scale of only a few tens of picoseconds.^24^ Efficient funneling of charge carriers may also be facilitated by the presence of built-in electric fields arising from the local difference in band gap between mixed-phase and iodide-rich domains, with such fields expected to increase in magnitude with increasing bromide fraction (Figure S4). Given that many factors affect the diffusion length of charge carriers in these materials, the funneling probability of any given charge carrier to an iodide-rich domain is unlikely to have a linear relationship with the rate with which the material volume segregates. One clear example of such a nonlinearity (illustrated in the right panel of Figure 3a) occurs when the diffusion length significantly exceeds the average distance between a photogenerated charge carrier and an iodide-rich domain. At this point, all charges will be captured into iodide-rich domains and no further change in average photon energy will be apparent even if further parts of the film continue to segregate over time (Figure 1). This effect is clearly apparent in the data presented in Figure 2 (a)-(c), and may also account for the PL-determined segregation rate generally being substantially faster than that determined from XRD for the full material volume (Figure 2d).^20^ A second example is a case for which a chemical treatment aimed to suppress halide segregation instead inadvertently reduces charge-carrier mobility or increases trap-mediated recombination. In this case, the charge-carrier diffusion length and therefore the PL recorded from iodide-rich domains will decrease, suggesting successful suppression, even if the dynamics of halide segregation have remained the same. As a result of such nonlinearities, the rate of PL red shift therefore does not directly reflect the rate with which the overall mixed-halide perovskite volume segregates.

Second, we show that the energy band offset between the mixed-halide and iodide-rich phases strongly influences the exchange of charges and therefore the PL spectra. As indicated schematically in Figure 3b, for small energy offsets (i.e., low bromide content), we would expect that a substantial fraction of charge carriers funneled into iodide-rich regions can thermally escape back into the mixed phase, given that relative charge-carrier populations are governed by Boltzmann statistics. For higher bromide fractions, on the other hand, the energy offset between the two phases becomes too large and charge carriers funneled into iodide-rich regions mostly recombine there, enhancing the red-shifted PL intensity.

These effects are clearly evident in the data presented in Figure 2, which reveals a transition occurring between the regimes of thermal escape and full localization for bromide fractions around *x* = 0.2. Figure 2e shows that the difference Δ*E* between the initial (*E*_I_) and terminal (*E*_T_) average PL photon energies is initially very small for bromide fractions between 0 and 0.2 (see also Figure S4 for a plot of *E*_I_ and *E*_T_). However, as Δ*E* exceeds the ambient thermal energy (26 meV) for *x* > 0.2, these energy shifts suddenly increase steeply with bromide content as charges are becoming fully localized in iodide-rich regions. In further evidence for such effects, we note that for *x* < 0.2 the segregation rates determined from PL and XRD experiments (*k*_⟨*E*⟩_ and *k*_XRD_) exhibit the same value (Figure 2d). For these low bromide fractions, effective thermal activation allows charge carriers to escape iodide-rich domains, respreading them across the full volume of the film. As a result, both measurements accurately reflect the full material evolution and return the same result. However, when Δ*E* becomes larger than thermal energies (for *x* > 0.2) photoluminescence spectroscopy and X-ray diffraction no longer probe the same material landscape and segregation rates returned by both techniques sharply diverge (Figure 2d). We note that these findings also suggest that the apparent threshold for halide segregation at a bromide fraction of 0.2 commonly invoked in the literature may simply be an experimental artifact arising from the sole use of such photoluminescence measurements.^20,23,25,26,41,70,71^ When techniques such as XRD are implemented that probe the full material volume across all compositions, such a threshold is in fact not observed.^20^

Third, charge funneling into iodide-rich domains changes the balance between radiative and nonradiative recombination pathways, which influences the perceived growth in red-shifted PL intensity (see schematic in Figure 3c). Nonradiative rates are affected by charge funneling because mixed iodide-bromide perovskites generally exhibit higher trap-state densities with increasing bromide fraction,^66^ partly resulting from the higher tendency of bromide-rich perovskites to form detrimental negatively charged bromine interstitials.^72–75^ We also observe this effect here: Figure 2f highlights how, with increasing bromide fraction, the PL intensity recorded for the initial mixed phase decreases. Hence, we propose that emerging iodide-rich regions exhibit a smaller trap-mediated recombination rate compared to that of the original mixed-phase material. In particular, a more bromide-rich initial stoichiometry would result in a larger enhancement in the photoluminescence intensity when the emitting material shifts to the iodide-rich terminal composition representing *x* ≈ 0.2.^3,7^ In addition, the radiative recombination rate will be strongly affected by the charge-carrier concentration effect. As charge-carriers funnel into the small volume occupied by iodide-rich material, the local charge-carrier density increases, which has been shown to cause enhanced second-order electron–hole recombination.^3,24^ This boost in radiative rate will be particularly effective for *x* > 0.2 for which charge carriers are strongly localized in iodide-rich domains with little possibility for thermal escape. Taken together, these changes in recombination dynamics mean that charge carriers in iodide-rich regions are far more likely to recombine radiatively than in their mixed-phase counterparts. This is exactly what is observed in Figure 2g, which reveals a strong relative boost in emission intensity following segregation, for compositions with bromide fraction larger than 0.2. Importantly, such boost in radiative efficiency will cause a fast red-shift in the PL spectra that is not representative of the amount of halide segregation occurring in the overall material volume. In addition, the use of passivating agents will manipulate the recombination kinetics of both the mixed and iodide-rich phase, leading to further changes in the kinetics of the PL red-shift.^69^

Fourth, in the presence of charge extraction layers, the balance between charge extraction and retransfer into the perovskite affects the magnitude of the PL intensity recorded from iodide-rich domains (see Figure 3d). Such dynamics may in turn lead to erroneous conclusions on the extent of halide segregation occurring. While we do not examine such effects here experimentally, we note for completeness that work by Lim et al.^69^ has evidenced how the presence of a hole-transport layer (HTL) such as poly­(triaryl amine) (PTAA) interfaced with a mixed-halide perovskite can affect the PL spectra recorded as the perovskite undergoes segregation. A much greater enhancement in iodide-rich PL emission is observed when the PTAA layer is added, despite in situ XRD measurements displaying reduced segregation.^69^ This apparent discrepancy arises from both the preferential formation of iodide-rich regions near the MHP-HTL interface, and favorable energetic alignment allowing efficient hole back-transfer from the HTL to the iodide-rich perovskite regions. As a result, the emission intensity from such iodide-rich regions is enhanced in the presence of a charge extraction layer even if the actual extent of halide segregation is minimal.^69^ In addition, charge-extraction in a full device structure will also influence segregation kinetics as probed via PL spectroscopy.^33^ While some charge can be extracted from iodide-rich regions via percolation pathways, extraction from such regions is far less effective than from the original mixed-phase perovskite.^33^ If charges are not extracted, they are more likely to recombine, thus yielding higher PL intensity from iodide-rich domains, which will return an artificially inflated segregation rate from PL measurements under operational device conditions (see right panel of Figure 3d).^33^ Finally, the trapping of charge carriers by certain defect species has been proposed as a driver of halide segregation, an effect that may be countered by timely charge extraction. Zhou et al. have indeed demonstrated that, as charge-extraction and charge-trapping occur on comparable time scales, illuminated perovskite films that appear unstable under open-circuit conditions can exhibit enhanced resistance to halide segregation under bias conditions.^30^ Such competition between charge-extraction and charge-trapping will however further influence how the PL spectrum evolves as the perovskite segregates.

In summary, while the development of methods to suppress halide segregation in mixed-halide perovskites is an essential goal toward their implementation in multijunction solar cells, monitoring the efficacy of such methods through changes in the photoluminescence spectra is not advisable. While frequently deployed to date,^9,42,44,46,47,51–54^ PL monitoring does not accurately reflect the rate or extent of halide segregation, because a myriad of other factors also affect the recombination kinetics of charge carriers. As we have shown, important factors include charge diffusion and capture into iodide-rich domains, thermal reactivation into the mixed phase, changes in nonradiative and radiative recombination pathways with halide content and modified extraction pathways in a device structure. As a result, the commonly monitored red shifts occurring in PL spectra during segregation do not reliably reflect the average material response across the full volume. Photoluminescence spectroscopy therefore at best provides a simple but sensitive binary measure of whether some amount of halide segregation has occurred. Before any sensible conclusions regarding the extent or dynamics of halide segregation could be drawn from photoluminescence measurements, the factors listed above would have to be properly accounted for, which would form a nontrivial task. Therefore, we suggest that all future studies investigating halide segregation supplement or replace photoluminescence spectroscopy with a technique that probes structural properties evenly across the bulk material. Suitable techniques include X-ray diffraction and optical absorption spectroscopy.^20,36,40,45,49,61,62,70,76–80^ Such approaches will ultimately allow for the effective development of segregation-resistant perovskite solar absorbers for efficient and photostable multijunction solar cells.

## Supplementary Material



## Notes

Sample fabrication details, experimental details of spectroscopic techniques, supplementary experimental data (PDF)
