# Supplementary material for: Is Photoluminescence Spectroscopy a Suitable Probe of Halide Segregation?
Source: ACS Energy Lett. 2026 Apr 21;11(5):3953–61. doi: 10.1021/acsenergylett.6c00432 (PMC13162306; doi:10.1021/acsenergylett.6c00432)
Supplement: Supplementary file 1 [file nz6c00432_si_001.pdf]

Supporting Information

Is Photoluminescence Spectroscopy a Suitable Probe of Halide Segregation?

Joshua R. S. Lilly[a], Vincent. J.-Y. Lim[a], Jay B. Patel[b], Jae Eun Lee[a], Siyu Yan[a], Michael B. Johnston[a], Laura M. Herz[,a]*

[a] Clarendon Laboratory, Department of Physics, University of Oxford, Parks Road, Oxford, OX1 3PU, United Kingdom

[b] Department of Physics, King's College London, Strand, London, WC2R 2LS, United Kingdom

Corresponding Author

*laura.herz@physics.ox.ac.uk

1 Experimental Details

As described in more detail previously,[1–3] a custom-built sample stage was used to obtain X-ray diffraction profiles under light soaking (in situ). The stage was mounted inside a Rigaku SmartLab X-ray diffractometer. Non-synchrotron-based XRD irradiation (under similar exposure times as utilised in this study) has been shown not to influence the material structure of $MAPb(I_{1-x}Br_x)_3$.[1,2] The Cu-Kα_1 line was used for measurements, with a 2D X-ray detector (HyPix-3000) allowing for continual acquisition across a fixed 2θ range. The (200) peak was selected to increase angular resolution, whilst still maintaining sufficient signal intensity. Thin films were illuminated with laser light from a fibre-coupled 470 nm diode laser (PicoQuant LDH-D-C-470). Routed via an optical fibre, laser light was launched into free space towards the thin film. The optical spot size was selected such that it was significantly larger than that of the X-ray probe beam.[1] The intensity at the centre of the optical excitation (i.e., the locality of the diffraction probe) was calculated to be 0.91 $mWcm^{-2}$.[1] Photoluminescence was recorded in parallel by collimation into a second optical fibre which was connected to an external Ocean Optics USB2000 spectrometer. In Figure 1a and Figure 1b a moving average window was applied along the wavelength axis to reduce the visual noise level; this moving average window had a width of approximately 15nm. Except during initial deposition, samples had never been previously exposed to light, were transferred to the diffractometer wrapped in foil, and the diffractometer was shielded from all other external light sources during the experiment.

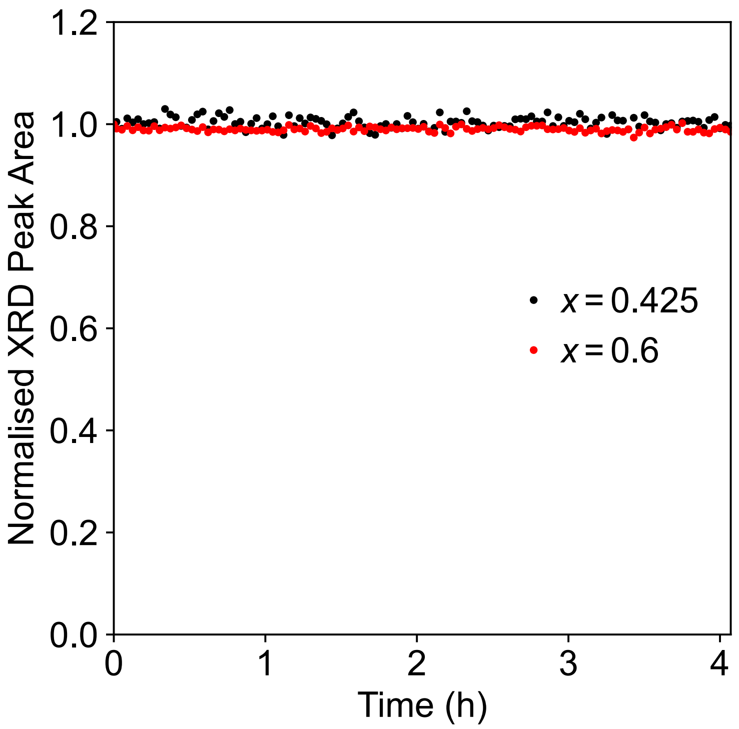

Figure S1. Area underneath the second-order diffraction peak for $MAPb(I_{1-x}Br_x)_3$ films with two different bromide content values *x*, normalised to the initial peak area. Films were encapsulated with PMMA and illuminated with a constant intensity of 0.91 $mWcm^{-2}$ via 470 nm continuous-wave laser excitation.

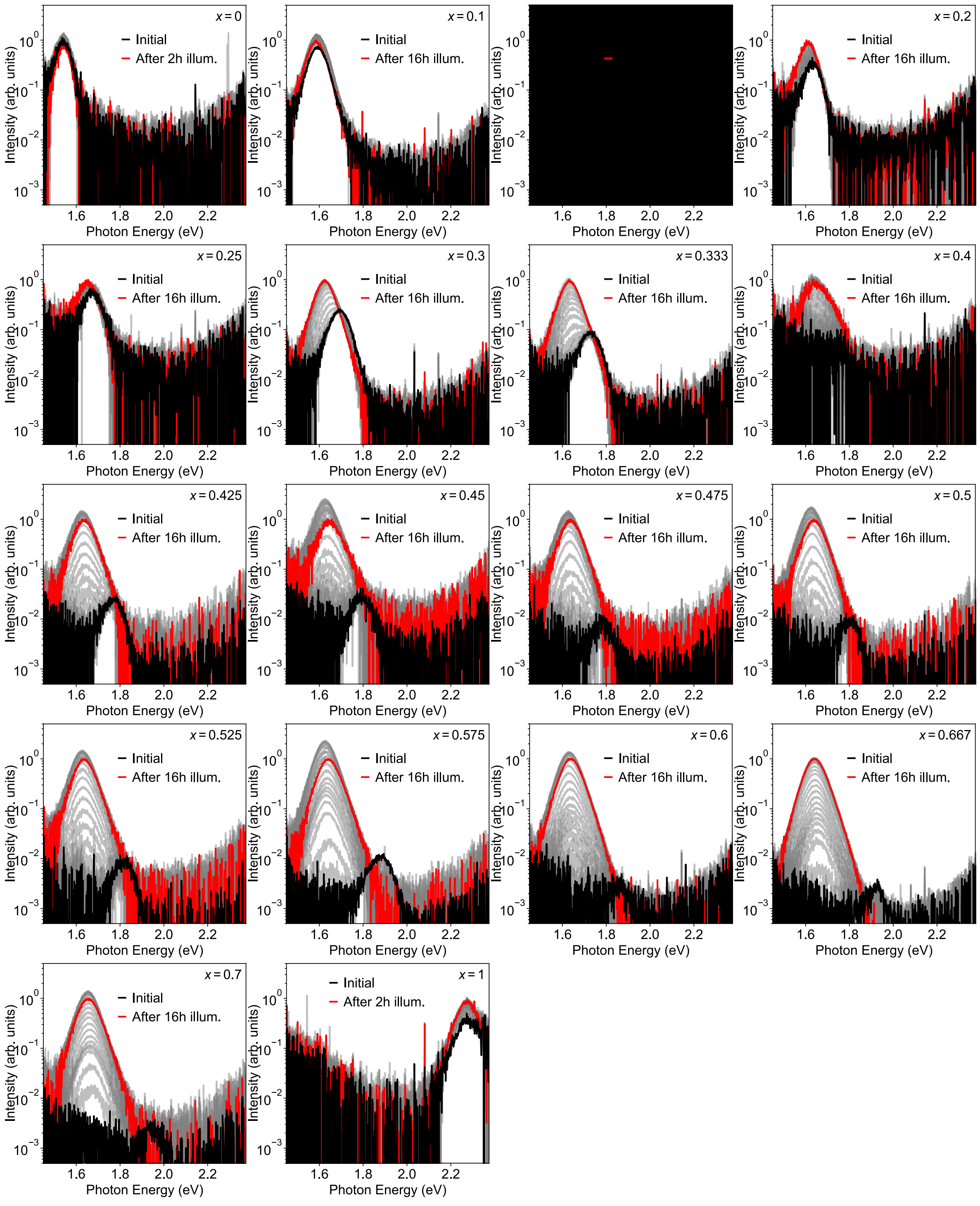

Figure S2. Photoluminescence spectra of $MAPb(I_{1-x}Br_x)_3$ films recorded at the experiment onset (black trace) and after 16 h of illumination (red trace). Grey traces indicate log-spaced intermediate spectra. Samples were encapsulated and illuminated with a constant intensity of 0.91 $mWcm^{-2}$ via a 470 nm laser excitation. Note, single-halide samples were only light-soaked for two hours, as they are not susceptible to halide segregation. The nominal bromide fraction is indicated in the upper-right of each individual plot.

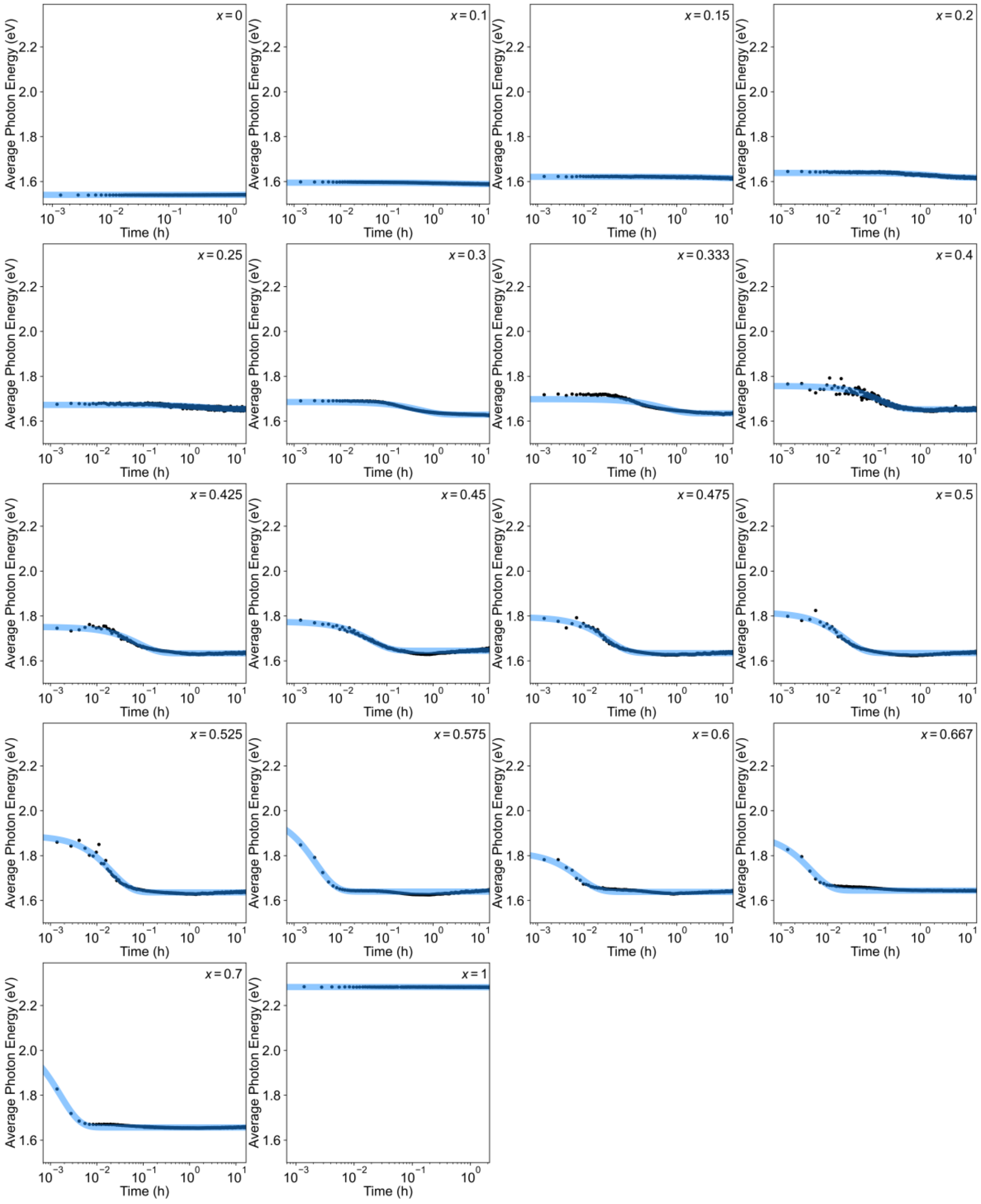

Figure S3. Average photon energy evolutions for $MAPb(I_{1-x}Br_x)_3$ films. The model fit is highlighted in blue. Samples were encapsulated and illuminated with a constant intensity of 0.91 mWcm^{-2} via a 470 nm laser excitation. Note, single-halide compositions were only light-soaked for two hours, as they are not susceptible to halide segregation. The nominal bromide fraction *x* is indicated in the upper-right of each individual plot. For mixed-halide samples, integrals were computed between 1.5 eV and 2.1 eV. For single-halide compositions, a 0.1 eV integrating window (centred on the time zero PL peak centre) was utilised to negate the impact of background fluctuations.

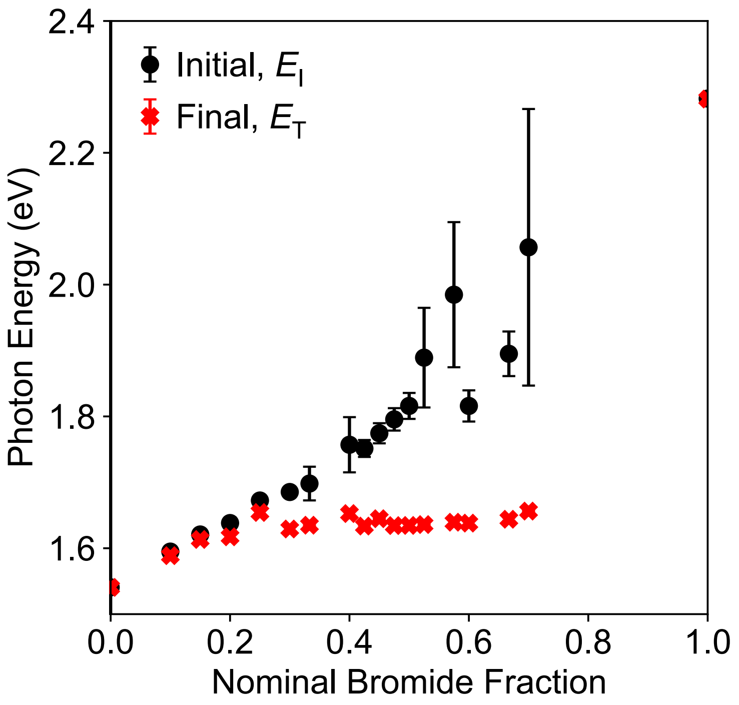

Figure S4. Initial (E_I, black circles) and terminal (E_T, red crosses) average photon energies extracted from the applied model (Figure S3) for $MAPb(I_{1-x}Br_x)_3$ films.

2 Sample Fabrication

$MAPb(I_{1-x}Br_x)_3$ thin films were prepared using the acetonitrile route as previously reported.[1,4,5] MAI (Greatcell), MABr (Greatcell), PbI_2 (TCI Chemicals 99.99%) and $PbBr_2$ (Thermo Scientific 98%) were weighed out and dissolved in a methylamine/acetonitrile (Merck Chemicals) solvent system to give a 0.5 M perovskite solution as described previously by Noel et al..[4] The solutions were then statically spin-coated onto z-cut quartz substrates (13 mm diameter) in a nitrogen filled drybox at 2000 rpm for 45 s. The films were then annealed at 100 °C for 90 minutes. PMMA (poly(methyl methacrylate), Sigma-Aldrich, mean molecular weight 120,000 g M^{-1}) was dissolved in chlorobenzene at 150 mg ml^{-1}, and then 75 µL of solution was statically deposited at a speed of 2000 rpm, 2000 rpm s^{-1} acceleration, for 30 s. After spin-coating of PMMA encapsulant, films were ready to use without additional annealing. Additional characterisation of these thin films has been presented previously.[1] Contact Stylus Profilometry (Dektak 150 Surface Profiler) measurements were completed on five unencapsulated films of varying bromide fraction (x = 0, 0.3, 0.5, 0.7, 1). All films measured exceeded 750 nm in thickness, consistent with previous reports employing the same fabrication methodology.[5] The nominal bromide fraction, determined from precursor stoichiometry, has been utilised to assign the composition of each film. Previous analysis of these films has shown that the change in diffraction peak angle with increasing bromide fraction is monotonic and linear (satisfying Vegard's law),[1] thus suggesting that there is negligible deviation of the actual halide ratio from the nominal halide ratio.

3 Calculating the Relative Structural Change

The relative structural change is a metric that parameterises how the X-ray diffraction pattern evolves over time.[1] This metric provides a measure of the relative proportion of material that has exhibited a change in lattice parameter, and hence is indicative of the volume of bromide/iodide-rich regions forming due to halide segregation. The complete mathematical formulation of this metric has been published previously.[1] In summary, the absolute integral of the relative differential intensity (i.e., the shaded regions above and below the x-axis in Figure 1d and Figure 1e) at any given illumination time is calculated, and is normalised with respect to the peak prior to illumination. Poisson statistics can be utilised to remove any base-line shift induced by the absolute integration of shot-to-shot noise.

A correction term (*B* term in the mathematical formulation) is applied in the calculation of the relative structural change metric that accounts for any change in the total integrated area of the tracked diffraction peak. Such changes in the total integrated area could arise due to film degradation (loss of crystallinity), X-ray flux fluctuations, or a change in the distribution of grain orientations. Figure S1 shows that the integrated diffraction peak intensity remains approximately constant throughout the experiment, suggesting negligible impact of the above effects.

In the $MAPb(Br_xI_{1-x})_3$ system, halide segregation causes the development of low-angle (iodide-rich regions) and high-angle (bromide-rich regions) wings in the diffraction peak (see Figure 1d and 1e). Due to the subsequently small angular region of interest, any change in the Lorentz-polarisation factor has been neglected in calculation of the segregation-induced relative structural change.[6] However, in the mixed-A-site-cation systems, halide segregation has been suggested to be preceded by A-site demixing, leading to more substantial peak shifts and possible changes in structural symmetry.[2] Consequently, in such scenarios, the relative structural change metric may no longer be representative of the amount of halide segregation that has occurred, and a more detailed analysis may be required.

Mathematically, the relative structural change (RSC) at time *t* can be described as:[1]

$$\mathrm{RSC}(t) = \left(\frac{\int |I(t,2\theta) - I(t=0,2\theta)|\mathrm{d}2\theta}{\int I(t=0,2\theta)\mathrm{d}2\theta} - \frac{|\int I(t,2\theta) - I(t=0,2\theta)\mathrm{d}2\theta|}{\int I(t=0,2\theta)\mathrm{d}2\theta}\right) - \left(\frac{\int |I(t,2\theta) - I'(t,2\theta)|\mathrm{d}2\theta}{\int I(t=0,2\theta)\mathrm{d}2\theta} - \frac{|\int I(t,2\theta) - I'(t,2\theta)\mathrm{d}2\theta|}{\int I(t=0,2\theta)\mathrm{d}2\theta}\right) = (A-B)-(C-D),$$

(1)

where *I* is the measured XRD intensity at an angle 2θ and time *t*, and *A*, *B*, *C*, and *D* are defined to allow for later referral to each of the terms in Eq. 1. *I'* is a synthetic XRD pattern that is generated by utilising a Poisson sample as such:

$$I'_n = \mathrm{Poisson}\left(\frac{\sum_{n+1}^{n+5} I_m + \sum_{n-5}^{n-1} I_m}{10}\right)$$

(2)

where *n* is the nth recorded XRD pattern (evenly spaced, with a total of *N* recorded XRD patterns). All integrals were evaluated between 27.5° and 31°. As *I'* is calculated via a ten-point effective rolling average (used to obtain a low-noise estimate from which to take the Poisson sample), there are ten time steps that do not have a correction directly calculated. *C* and *D* are set to zero for the initial (t = 0) time step, are taken as an average of time steps six through ten to fill in the second through fifth time step, and are taken as an average of the $(N-9)^{\mathrm{th}}$ through to the $(N-5)^{\mathrm{th}}$ time step for the final five points. Example relative structural change traces, illustrating the correction procedure, have been shown previously.[1]

In Eq. 1, the first term (*A*) calculates the absolute change in the second-order peak with respect to the time-zero peak (shading, Figure 1d and 1e). The second term (*B*) corrects for any change in integrated peak area that is not associated with halide segregation. The third (*C*) and fourth (*D*) term (copying the structure of the first two terms) correct for the noise that is integrated in *A* and *B* (as an absolute integral is taken, normally distributed noise will no longer sum to zero). *D* will effectively always go to zero, but is included to ensure mathematical consistency.

X-rays can readily penetrate several microns deep in perovskite films.[7] On the other hand, for photon energies significantly above band gap (470nm laser excitation), the optical penetration depth can be much shorter (on the order of 50nm for $MAPbI_3$).[8] Crucially, however, excited charge carriers have been shown to readily diffuse through several hundred nanometers of metal-halide perovskite within 1 ns.[8,9] Given that charge-carrier lifetimes are typically much longer than 1 ns, charge carriers are expected to be spread quite evenly across the films depth

profile when they recombine and emit light. Therefore, substantial differences in the probing depths of XRD and PL spectroscopy are not expected.

In addition, our adoption of the relative structural change metric (RSC) is a powerful tool to unravel the impact solely related to material that does segregate. Even if a small region had low charge-carrier density and therefore negligible halide segregation, the relative structural change, as a differential metric, means that the dynamics and the extracted rate of segregation will not be affected.

4 Additional XRD Analysis

X-ray diffraction taken in situ has previously been presented by Lilly *et al.*;[1] here we provide further discussion and analysis. Figure S5, Figure S6, and Figure S7 show the development of the second-order diffraction peak, corresponding differential intensity profiles, and the evolution of the relative structural change respectively for all measured compositions.[1] For single-halide compositions, there can be no halide segregation, and thus, the relative structural change should be effectively zero: this is exactly what is observed (Figure S7).

Previous work has observed symmetric broadening of the diffraction peak under illumination for $MAPb(Br_{0.5}I_{0.5})_3$.[2] Rather than being attributed to material microstrain, the work of Suchan *et al.* indicated that the broadened peak is a result of the light-induced formation of a broad range of different halide compositions (i.e., halide segregation).[10] As shown in Figure S8 and Figure S9, we observe that whilst the peak centre of mass remains approximately constant throughout light-soaking for all compositions, mixed-halide films exhibit a monotonic increase in peak breadth.[11] The total integrated peak intensity is shown to be approximately constant (see Figure S10) suggesting minimal material conversion to amorphous phases or constituent elemental loss.[12] The increase in peak breadth is represented in the calculation of the relative structural change metric. As $MAPb(Br_xI_{1-x})_3$ has been demonstrated to follow Vegard's law,[1] an unchanging peak centre of mass suggests that the as-formed iodide-rich and bromide-rich regions are of similar crystalline quality and that there is negligible change in the net strain of the system.[10]

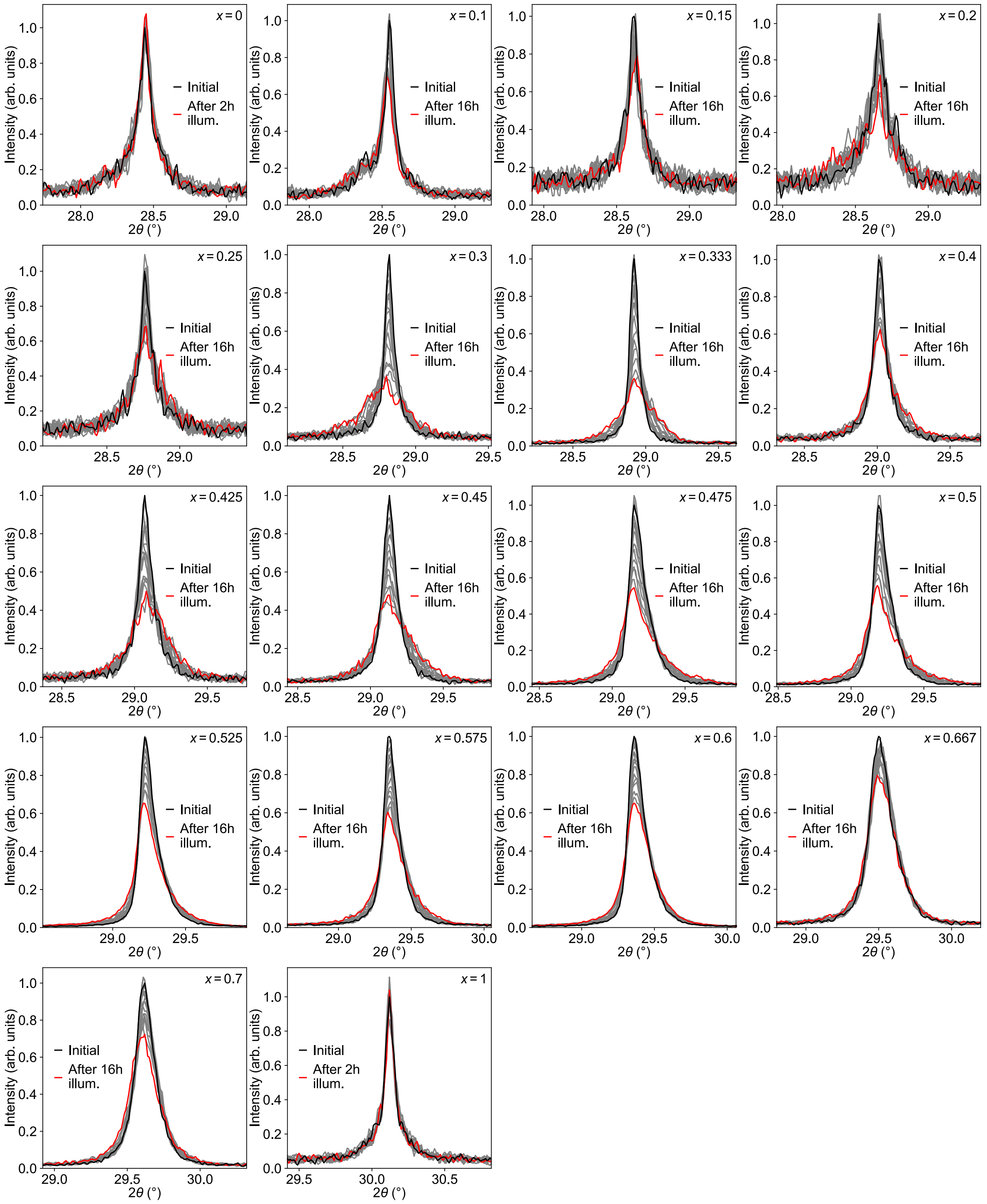

Figure S5. Evolution of the second-order diffraction peak of $MAPb(I_{1-x}Br_x)_3$ films recorded at the experiment onset (black trace) and after 16 h (2 h for single-halide films) of illumination (red trace). Grey traces indicate log-spaced intermediate measurements taken at the same time points as those in Figure S2. Films were encapsulated and illuminated with a constant intensity of 0.91 mWcm^{-2} via a 470 nm laser excitation. The nominal bromide fraction is indicated in the upper-right of each individual plot.

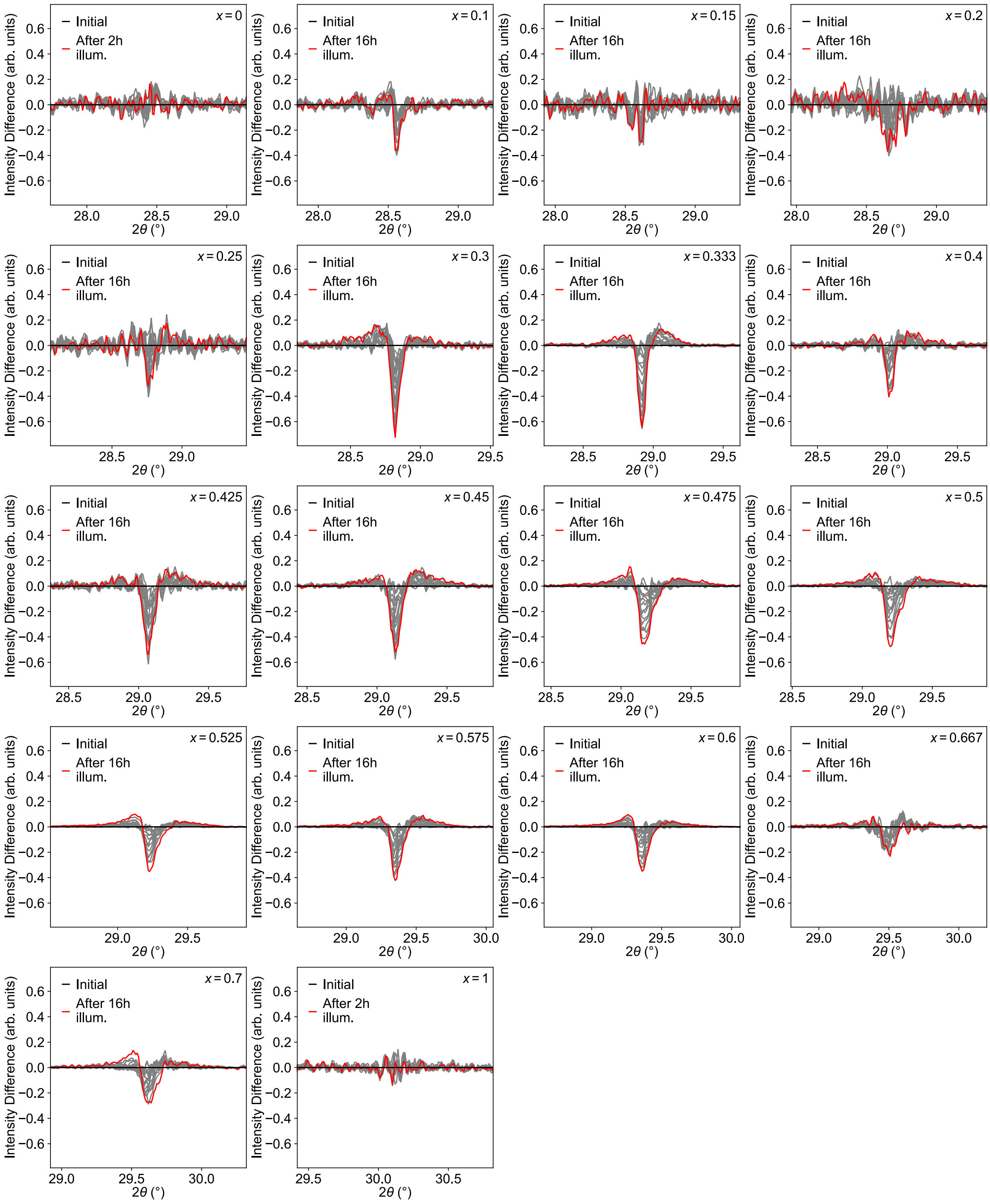

Figure S6. Evolution of the differential diffraction signal of $MAPb(I_{1-x}Br_x)_3$ films recorded at the experiment onset (black trace) and after 16 h (2 h for single-halide films) of illumination (red trace). Grey traces indicate log-spaced intermediate measurements taken at the same time points as those in Figure S5. Films were encapsulated and illuminated with a constant intensity of 0.91 $mWcm^{-2}$ via a 470 nm laser excitation. The nominal bromide fraction is indicated in the upper-right of each individual plot.

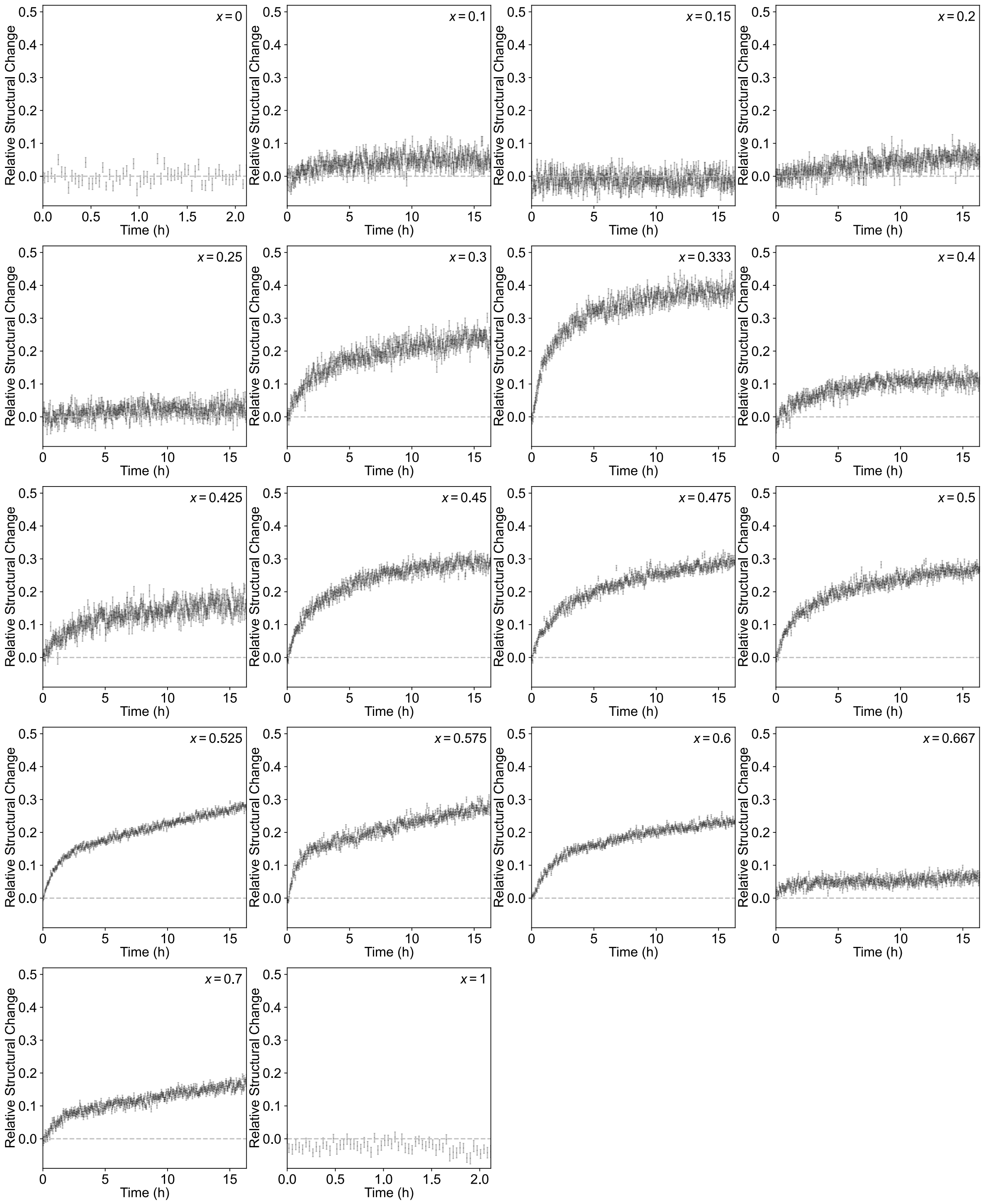

Figure S7. Relative structural change in thin films of $MAPb(I_{1-x}Br_x)_3$ arising from halide segregation. The relative structural change is calculated by taking the absolute integral of the differential X-ray diffraction intensity (Figure S6), and normalising with respect to the time-zero diffraction profile (further details in Section 3 of the Supporting Information). Films were encapsulated and illuminated with a constant intensity of 0.91 $mWcm^{-2}$ via 470 nm laser excitation. The nominal bromide fraction is indicated in the upper-right of each individual plot.

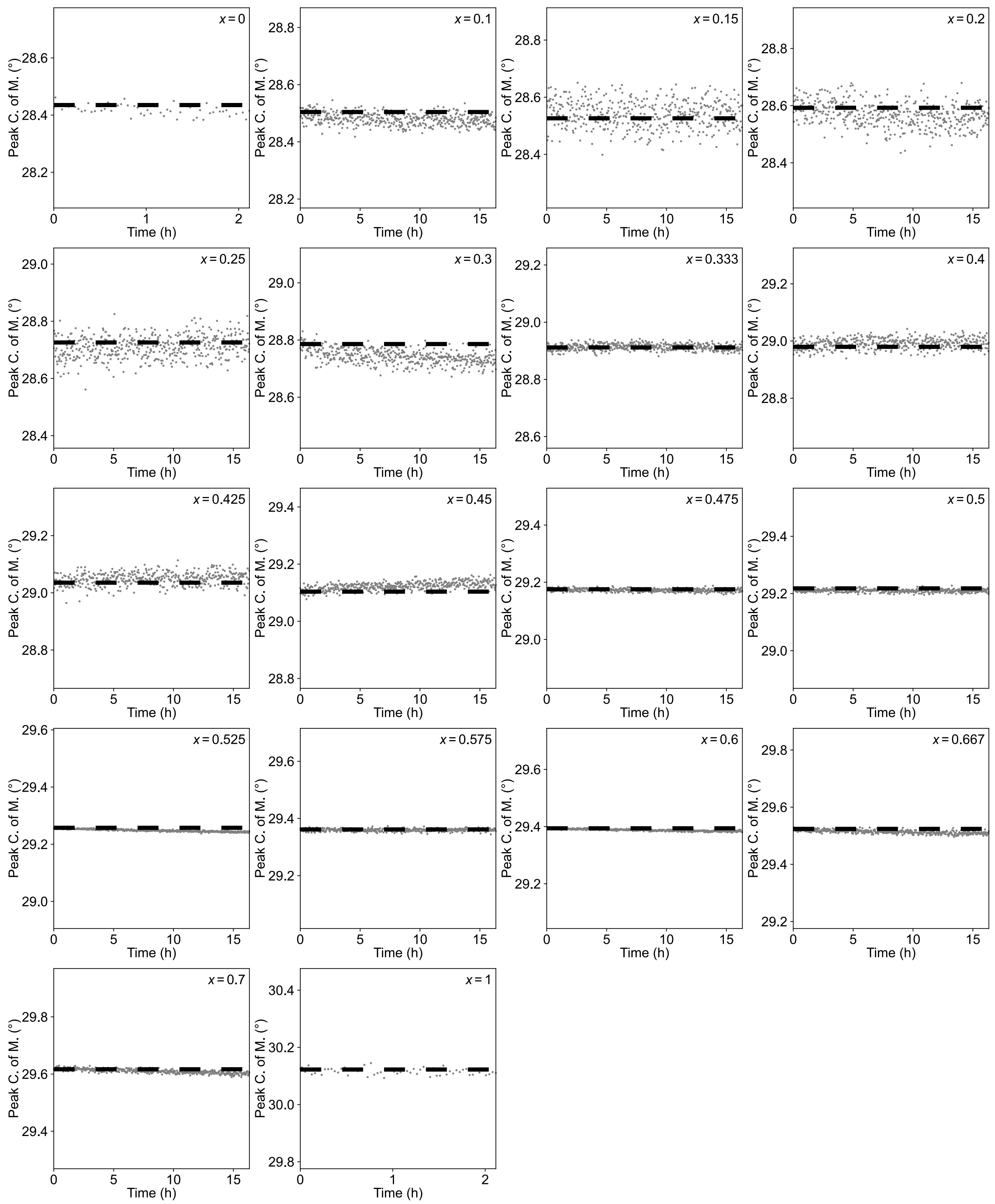

Figure S8. Evolution of the second-order diffraction peak centre of mass for $MAPb(I_{1-x}Br_x)_3$ films. The dashed line is set at the average peak centre of mass for the first five time steps. Films were encapsulated and illuminated with a constant intensity of 0.91 mWcm^{-2} via a 470 nm laser excitation. The nominal bromide fraction is indicated in the upper-right of each individual plot.

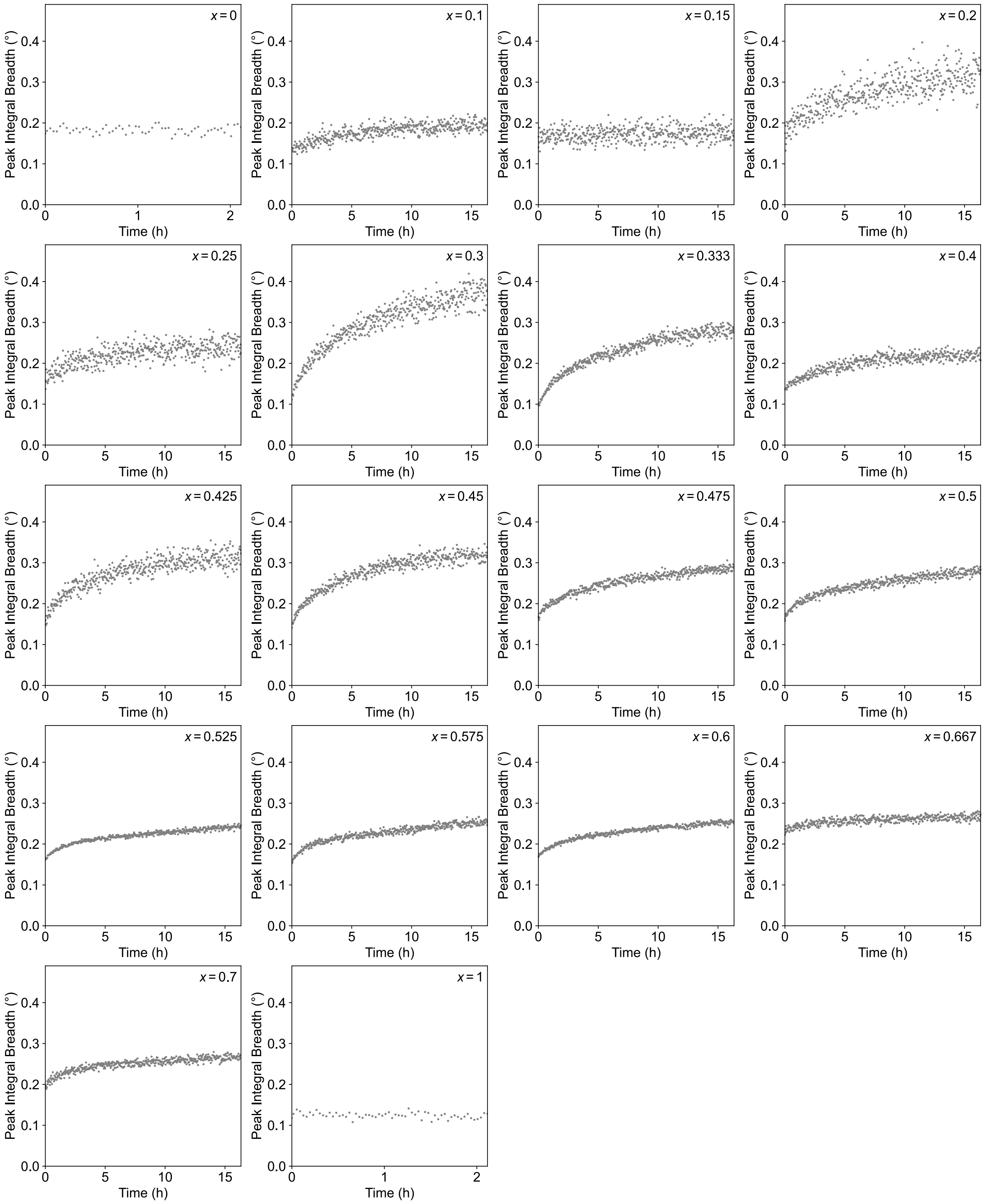

Figure S9. Evolution of the second-order diffraction peak integral breadth for $MAPb(I_{1-x}Br_x)_3$ films.[11] Films were encapsulated and illuminated with a constant intensity of 0.91 mWcm^{-2} via a 470 nm laser excitation. The nominal bromide fraction is indicated in the upper-right of each individual plot.

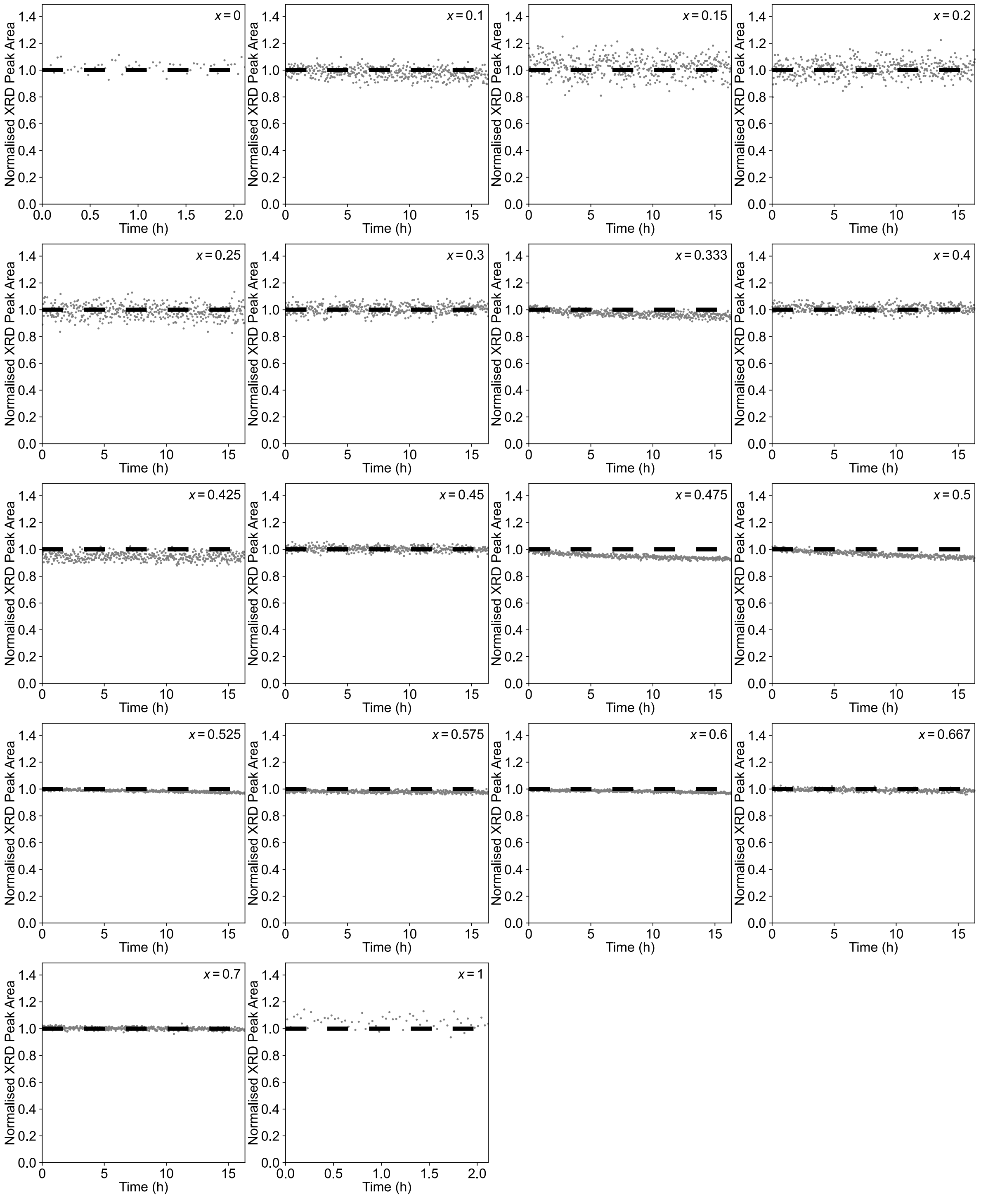

Figure S10. Evolution of the second-order diffraction peak integrated area for $MAPb(I_{1-x}Br_x)_3$ films normalised to the time-zero value. Films were encapsulated and illuminated with a constant intensity of 0.91 $mWcm^{-2}$ via a 470 nm laser excitation. The nominal bromide fraction is indicated in the upper-right of each individual plot.

5 Comparing Ensemble and Spatially-Resolved Measurements of Halide Segregation

PL spectroscopy is the most widely utilised tool in the MHP-device community to assess halide segregation in mixed-halide films. As halide segregation is a stochastic process, varying on a grain-to-grain scale (and even within grains),[13–15] accurate assessment of compositional dependencies requires an ensemble measurement that is able to average across bulk films. Furthermore, to assess the dynamics of halide segregation, it is essential that in situ measurements can be completed (as halide segregation will reverse upon cessation of light-soaking). Such ensemble in situ measurements can be facilitated with the use of synchronous XRD and PL spectroscopy under continuous illumination, as presented here.

From our in situ PL/XRD measurements, we illustrate in Figure 3 how in a segregated film, excited charge carriers funnel, localise, and radiatively recombine in iodide-rich regions of perovskite. This conclusion is supported by the highly localised radiative emission from iodide-rich regions (on the order of 100 nm in extent) as identified with cathode luminescence imaging by Bischak *et al.*[13] These small, narrow-bandgap iodide-rich domains, will readily localise change carriers, leading to an increase in the local charge-carrier density, and hence resulting in a boosted radiative efficiency.[16] Such within-grain segregation has further been demonstrated via cryo-electron-energy-loss spectroscopy by Fan *et al*. who showed the distinct formation of iodide-rich regions on a similar length scale.[15] Finally, Datta *et al*. evidenced how a red-shift in the PL spectrum (from hyperspectral imaging) correlated with an increase in local iodide-content as identified from TOF-SIMS measurements.[14]

Spatially-resolved measurements reported in the literature therefore also suggest that generated charge carriers rapidly localise into small (on the order of 100nm in extent) regions of iodide-rich perovskite. Such rapid funneling leads to the photoluminescence emission of the film being dominated by radiative recombination originating from such iodide-rich domains. Consequently, as outlined in Figure 3, changes in the photoluminescence spectrum observed during halide segregation are not representative of the underlying ionic re-arrangement. As a result, PL spectroscopy is not a suitable technique to assess the rate or extent of halide segregation that has occurred in a mixed-halide film.

References

[1] J. R. S. Lilly, V. J.-Y. Lim, J. B. Patel, S. Yan, J. E. Lee, M. B. Johnston, L. M. Herz, Impact of Halide Alloying on the Phase Segregation of Mixed-Halide Perovskites, *Small Struct.* **2026**, *7*, e202500545.

[2] A. J. Knight, J. Borchert, R. D. J. Oliver, J. B. Patel, P. G. Radaelli, H. J. Snaith, M. B. Johnston, L. M. Herz, Halide Segregation in Mixed-Halide Perovskites: Influence of A-Site Cations, *ACS Energy Lett.* **2021**, *6*, 799.

[3] V. J.-Y. Lim, A. J. Knight, R. D. J. Oliver, H. J. Snaith, M. B. Johnston, L. M. Herz, Impact of Hole-Transport Layer and Interface Passivation on Halide Segregation in Mixed-Halide Perovskites, *Adv. Funct. Mater.* **2022**, *32*, 2204825.

[4] N. K. Noel, S. N. Habisreutinger, B. Wenger, M. T. Klug, M. T. Hörantner, M. B. Johnston, R. J. Nicholas, D. T. Moore, H. J. Snaith, A Low Viscosity, Low Boiling Point, Clean Solvent System for the Rapid Crystallisation of Highly Specular Perovskite Films, *Energy Environ. Sci.* **2017**, *10*, 145.

[5] A. J. Knight, J. B. Patel, H. J. Snaith, M. B. Johnston, L. M. Herz, Trap States, Electric Fields, and Phase Segregation in Mixed-Halide Perovskite Photovoltaic Devices, *Adv. Energy Mater.* **2020**, *10*, 1903488.

[6] W. Yinghua, Lorentz–Polarization Factor for Correction of Diffraction-Line Profiles, *J. Appl. Cryst.* **1987**, *20*, 258.

[7] M. Qin, H. Xue, H. Zhang, H. Hu, K. Liu, Y. Li, Z. Qin, J. Ma, H. Zhu, K. Yan, G. Fang, G. Li, U.-S. Jeng, G. Brocks, S. Tao, X. Lu, Precise Control of Perovskite Crystallization Kinetics via Sequential A-Site Doping, *Advanced Materials* **2020**, *32*, 2004630.

[8] T. W. Crothers, R. L. Milot, J. B. Patel, E. S. Parrott, J. Schlipf, P. Müller-Buschbaum, M. B. Johnston, L. M. Herz, Photon Reabsorption Masks Intrinsic Bimolecular Charge-Carrier Recombination in CH3NH3PbI3 Perovskite, *Nano Lett.* **2017**, *17*, 5782.

[9] H. P. Pasanen, P. Vivo, L. Canil, H. Hempel, T. Unold, A. Abate, N. V. Tkachenko, Monitoring Charge Carrier Diffusion across a Perovskite Film with Transient Absorption Spectroscopy, *J. Phys. Chem. Lett.* **2020**, *11*, 445.

[10] K. Suchan, J. Just, P. Beblo, C. Rehermann, A. Merdasa, R. Mainz, I. G. Scheblykin, E. Unger, Multi-Stage Phase-Segregation of Mixed Halide Perovskites under Illumination: A Quantitative Comparison of Experimental Observations and Thermodynamic Models, *Adv. Funct. Mater.* **2023**, *33*, 2206047.

[11] W. L. Tan, C. R. McNeill, X-Ray Diffraction of Photovoltaic Perovskites: Principles and Applications, *Appl. Phys. Rev.* **2022**, *9*, 021310.

[12] Y. Zhou, S. C. W. van Laar, D. Meggiolaro, L. Gregori, S. Martani, J.-Y. Heng, K. Datta, J. Jiménez-López, F. Wang, E. L. Wong, I. Poli, A. Treglia, D. Cortecchia, M. Prato, L. Kobera, F. Gao, N. Zhao, R. A. J. Janssen, F. De Angelis, A. Petrozza, How Photogenerated I_2 Induces I-Rich Phase Formation in Lead Mixed Halide Perovskites, *Adv. Mater.* **2024**, *36*, 2305567.

[13] C. G. Bischak, C. L. Hetherington, H. Wu, S. Aloni, D. F. Ogletree, D. T. Limmer, N. S. Ginsberg, Origin of Reversible Photoinduced Phase Separation in Hybrid Perovskites, *Nano Lett.* **2017**, *17*, 1028.

[14] K. Datta, S. C. W. van Laar, M. Taddei, J. Hidalgo, T. Kodalle, G. J. W. Aalbers, B. Lai, R. Li, N. Tamura, J. T. W. Frencken, S. V. Quiroz Monnens, R. J. E. Westbrook, D. J. Graham, C. M. Sutter-Fella, J.-P. Correa-Baena, D. S. Ginger, M. M. Wienk, R. A. J. Janssen, Local Halide Heterogeneity Drives Surface Wrinkling in Mixed-Halide Wide-Bandgap Perovskites, *Nat Commun* **2025**, *16*, 1967.

[15] Q. Fan, Y. Cui, Y. Li, J. A. Vigil, Z. Jiang, P. Nandi, R. Colby, C. Zhang, Y. Cui, H. I. Karunadasa, A. M. Lindenberg, Phase Segregation Dynamics in Mixed-Halide Perovskites Revealed by Plunge-Freeze Cryo-Electron Microscopy, *Cell Rep. Phys. Sci.* **2025**, *6*, 102653.
[16] L. M. Herz, Charge-Carrier Dynamics in Organic-Inorganic Metal Halide Perovskites, *Annu. Rev. Phys. Chem.* **2016**, *67*, 65.
